# Bis[μ-2-(4-hydroxyphenyl)acetato]-κ^3^ *O*,*O*′:*O*;κ^3^ *O*:*O*,*O*′-bis{aqua(4,4′-bi­pyri­dine-κ*N*)bis[2-(4-hydroxyphenyl)acetato-κ^2^ *O*,*O*′]gadolinium(III)} monohydrate

**DOI:** 10.1107/S1600536810041255

**Published:** 2010-10-23

**Authors:** Jia-Lu Liu, Jian-Feng Liu, Guo-Liang Zhao

**Affiliations:** a College of Chemistry and Life Sciences, Zhejiang Normal University, Jinhua 321004, People’s Republic of China;, Zhejiang Normal University Xingzhi College, Jinhua 321004, People’s Republic of China

## Abstract

In the dinuclear title complex, [Gd_2_(C_8_H_7_O_3_)_6_(C_10_H_8_N_2_)_2_(H_2_O)_2_]·H_2_O, the two Gd^III^ ions are nine-coordinated by seven O atoms from four deprotonated *p*-hy­droxy­phenyl­acetic acid (PAA) ligands, one water O atom and an N atom from a 4,4′-bypyridine (bipy) ligand in a distorted tricapped trigonal-prismatic geometry. The deprotonated PAA ligands are coordinated to the Gd^III^ atom either as chelating on the same metal or in a tridentate bridging mode. Numerous O—H⋯O and O—H⋯N hydrogen bonds involving hydroxyl, coordinated and uncoordinated water mol­ecules build up an intricate three-dimensional network.

## Related literature

For the properties of carb­oxy­lic metal–organic complexes, see: Fang & Zhang (2006 ▶); Liu *et al.* (2010 ▶); Wang *et al.* (2010 ▶); Wang & Sevov (2008 ▶). For related structures, see: Favas *et al.* (1980 ▶); Hatscher (2005 ▶); John & Urland (2006 ▶). 
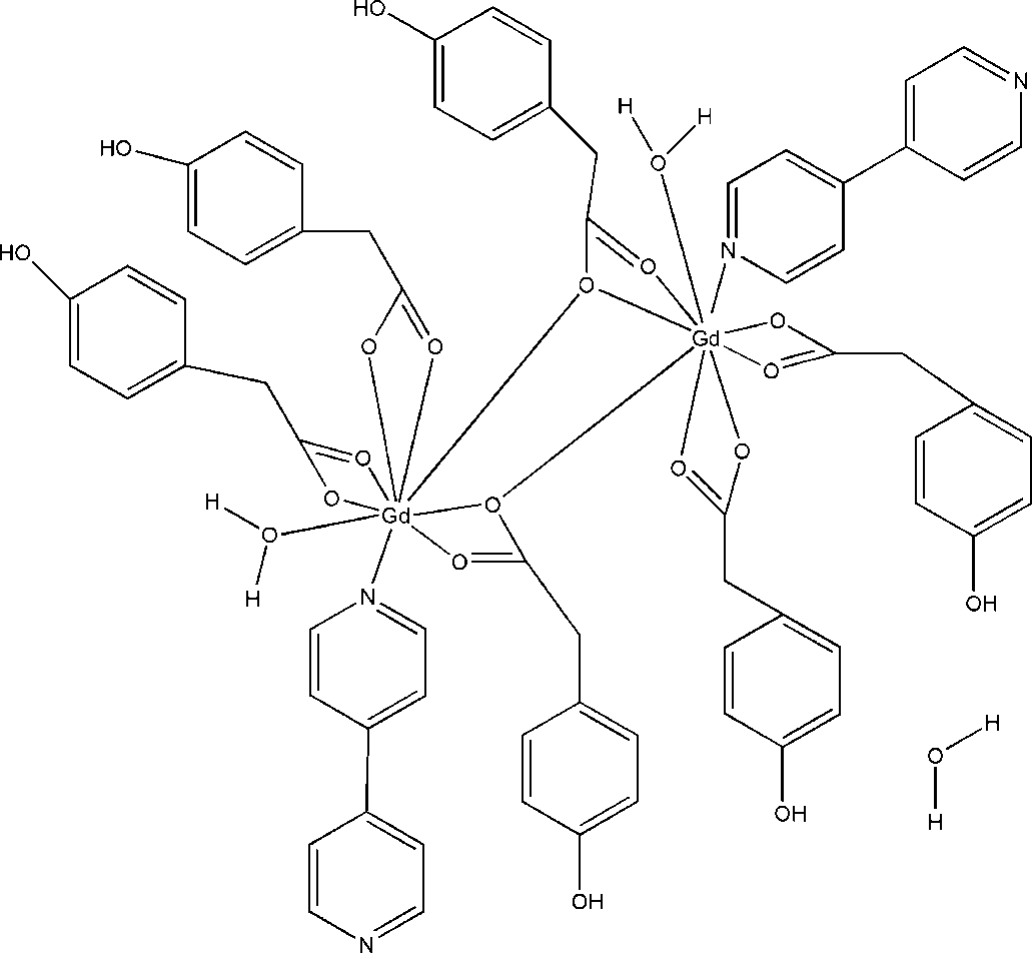


## Experimental

### 

#### Crystal data

[Gd_2_(C_8_H_7_O_3_)_6_(C_10_H_8_N_2_)_2_(H_2_O)_2_]·H_2_O
*M* *_r_* = 1587.73
Triclinic, 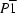

*a* = 11.7436 (1) Å
*b* = 16.2654 (2) Å
*c* = 18.4311 (2) Å
α = 83.52 (1)°
β = 72.11 (1)°
γ = 71.10 (1)°
*V* = 3169.4 (3) Å^3^
*Z* = 2
Mo *K*α radiation
μ = 2.16 mm^−1^
*T* = 296 K
0.15 × 0.13 × 0.12 mm

#### Data collection

Bruker APEXII area-detector diffractometer
Absorption correction: multi-scan (*SADABS*; Sheldrick, 1996 ▶) *T*_min_ = 0.731, *T*_max_ = 0.772
41430 measured reflections
11119 independent reflections
9631 reflections with *I* > 2σ(*I*)
*R*_int_ = 0.026

#### Refinement

*R*[*F* ^2^ > 2σ(*F* ^2^)] = 0.020
*wR*(*F* ^2^) = 0.048
*S* = 1.05
11117 reflections
862 parameters
H-atom parameters constrained
Δρ_max_ = 0.45 e Å^−3^
Δρ_min_ = −0.43 e Å^−3^

### 

Data collection: *APEX2* (Bruker, 2006 ▶); cell refinement: *SAINT* (Bruker, 2006 ▶); data reduction: *SAINT*; program(s) used to solve structure: *SHELXS97* (Sheldrick, 2008 ▶); program(s) used to refine structure: *SHELXL97* (Sheldrick, 2008 ▶); molecular graphics: *ORTEPIII* (Burnett & Johnson, 1996 ▶) and *ORTEP-3 for Windows* (Farrugia, 1997 ▶); software used to prepare material for publication: *SHELXL97*.

## Supplementary Material

Crystal structure: contains datablocks I, global. DOI: 10.1107/S1600536810041255/dn2609sup1.cif

Structure factors: contains datablocks I. DOI: 10.1107/S1600536810041255/dn2609Isup2.hkl

Additional supplementary materials:  crystallographic information; 3D view; checkCIF report

## Figures and Tables

**Table 1:** Hydrogen-bond geometry (Å, °)

*D*—H⋯*A*	*D*—H	H⋯*A*	*D*⋯*A*	*D*—H⋯*A*
O1*W*—H1*WB*⋯O13	0.77	2.00	2.744 (2)	161
O1*W*—H1*WA*⋯N4^i^	0.78	2.02	2.784 (3)	167
O2*W*—H2*WB*⋯O1	0.82	2.00	2.750 (2)	151
O2*W*—H2*WA*⋯N2^ii^	0.83	2.03	2.842 (3)	167
O3—H3⋯O3*W*	0.82	1.86	2.641 (3)	160
O6—H6⋯O12^i^	0.82	1.94	2.743 (3)	168
O9—H9⋯O17^iii^	0.82	1.87	2.675 (3)	167
O12—H12⋯O11^iv^	0.82	1.94	2.750 (3)	167
O15—H15⋯O3^v^	0.82	1.90	2.717 (3)	174
O18—H18⋯O9^ii^	0.82	1.97	2.767 (3)	162
O3*W*—H3*WA*⋯O4^vi^	0.81	1.96	2.773 (3)	179
O3*W*—H3*WB*⋯O6^ii^	0.83	1.99	2.808 (3)	165

Symmetry codes: (i) 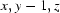; (ii) 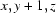; (iii) 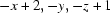; (iv) 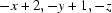; (v) 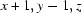; (vi) 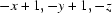.

**Fig. 1.**
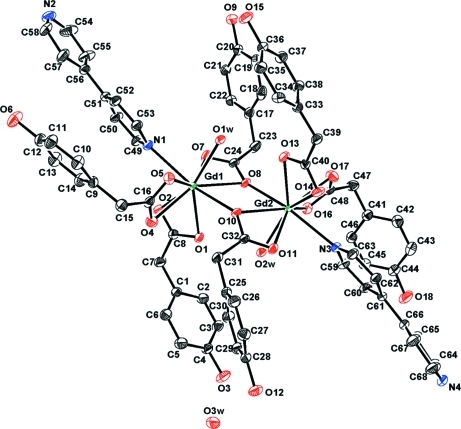
The molecular structure of the title complex, showing the atom-labelling scheme. Displacement ellipsoids are drawn at the 30% probability level. H atoms have been omited for the sake of clarity.

## supplementary crystallographic information

### Comment

The design and synthesis of carboxylic metal-organic complexes have attracted increasing interest for decades owing to their potential practical applications, such as fluorescence, magnetism (Wang, *et al.*, 2010; Fang, *et al.*, 2006; Wang, *et al.*, 2008). As part of our interest in this field (Liu, *et al.*, 2010), we report here the crystal structure of a new gadolinium(III) complex with the ligand *p*-hydroxyphenylacetic acid.

In the dinuclear title complex, the two Gd^III^ ions are nine coordinated by four *p*-hydroxyphenylacetic acid(PAA) ligands *via* seven O atoms, one O atom from water molecule and a N atom from bipy ligand in a distorted tricapped trigonal-prismatic geometry. Futhermore, the asymmetric unit contains one solvent water molecules (Fig. 1). Bond lengths and bond angles involving the metals and O atoms skeleton compare well with related structure as bis((µ2-Acetato-O,O,O')-diaqua-bis(acetato-O,O')-gadolinium(iii)) tetrahydrate (Favas *et al.*, 1980), bis(µ2-Acetato-O,O,O')-tetrakis(acetato-O,O')-tetra-aqua-di-gadolinium tetrahydrate (Hatscher, 2005) or bis(\m2-Acetato)-tetrakis(acetato)-diaqua-bis(4-pyridyloxy)-di-gadolinium dihydrate (John & Urland, 2006).

Numerous O-H···O hydrogen bonds involving hydroxyl, coordinated and non-coordinated water molecules, build up an intricated three dimensionnal network (Table 1).

### Experimental

All reagents and solvents were of commercially available quality and were used without further purification. P-hydroxyphenylacetic acid(HPAA)(3mmol) and sodium hydroxide (3mmol) were mixed together in water(10ml), then Gd[(NO_3_)_3_](1mmol) dissolved in water(10ml) was added into the above solution. After stirring for one hour, an ethanol(5ml) solution of 4,4'-bipyridine(1 mmol) was slowly dropped into the above solution with stirring for three hours. After filtration, the filtrate was allowed to stand at room temperature, and single crystals suitable for X-ray work were obtained after a week.

### Refinement

All H atoms attached to C atoms and O(hydroxyl) atom were fixed geometrically and treated as riding with C—H = 0.97 Å (methylene) or 0.93 Å (aromatic) and O—H = 0.82 Å with U_iso_(H) = 1.2U_eq_(C) or U_iso_(H) = 1.5U_eq_(O). H atoms of water molecule were located in a difference Fourier map and included in the subsequent refinement using restraints (O-H= 0.82 (1)Å and H···H= 1.39 (2)Å) with U_iso_(H) = 1.5U_eq_(O). In the last cycles of refinement they were treated as riding on their parent O atom.

### Crystal data

**Table:**

[Gd_2_(C_8_H_7_O_3_)_6_(C_10_H_8_N_2_)_2_(H_2_O)_2_]·H_2_O	*Z* = 2
*M**_r_* = 1587.73	*F*(000) = 1592
Triclinic, *P*1	*D*_x_ = 1.664 Mg m^−^^3^
Hall symbol: -P 1	Mo *K*α radiation, λ = 0.71073 Å
*a* = 11.7436 (1) Å	Cell parameters from 9500 reflections
*b* = 16.2654 (2) Å	θ = 1.2–25.0°
*c* = 18.4311 (2) Å	µ = 2.16 mm^−^^1^
α = 83.52 (1)°	*T* = 296 K
β = 72.11 (1)°	Block, colourless
γ = 71.10 (1)°	0.15 × 0.13 × 0.12 mm
*V* = 3169.4 (3) Å^3^	

### Data collection

**Table:**

Bruker APEXII area-detector diffractometer	11119 independent reflections
Radiation source: fine-focus sealed tube	9631 reflections with *I* > 2σ(*I*)
graphite	*R*_int_ = 0.026
φ and ω scans	θ_max_ = 25.0°, θ_min_ = 1.2°
Absorption correction: multi-scan (*SADABS*; Sheldrick, 1996)	*h* = −13→13
*T*_min_ = 0.731, *T*_max_ = 0.772	*k* = −19→18
41430 measured reflections	*l* = −21→21

### Refinement

**Table:**

Refinement on *F*^2^	Primary atom site location: structure-invariant direct methods
Least-squares matrix: full	Secondary atom site location: difference Fourier map
*R*[*F*^2^ > 2σ(*F*^2^)] = 0.020	Hydrogen site location: inferred from neighbouring sites
*wR*(*F*^2^) = 0.048	H-atom parameters constrained
*S* = 1.05	*w* = 1/[σ^2^(*F*_o_^2^) + (0.0216*P*)^2^ + 0.9907*P*] where *P* = (*F*_o_^2^ + 2*F*_c_^2^)/3
11117 reflections	(Δ/σ)_max_ = 0.003
862 parameters	Δρ_max_ = 0.45 e Å^−^^3^
0 restraints	Δρ_min_ = −0.43 e Å^−^^3^

### Special details

**Table:**

Geometry. All esds (except the esd in the dihedral angle between two l.s. planes) are estimated using the full covariance matrix. The cell esds are taken into account individually in the estimation of esds in distances, angles and torsion angles; correlations between esds in cell parameters are only used when they are defined by crystal symmetry. An approximate (isotropic) treatment of cell esds is used for estimating esds involving l.s. planes.
Refinement. Refinement of *F*^2^ against ALL reflections. The weighted *R*-factor *wR* and goodness of fit *S* are based on *F*^2^, conventional *R*-factors *R* are based on *F*, with *F* set to zero for negative *F*^2^. The threshold expression of *F*^2^ > σ(*F*^2^) is used only for calculating *R*-factors(gt) *etc*. and is not relevant to the choice of reflections for refinement. *R*-factors based on *F*^2^ are statistically about twice as large as those based on *F*, and *R*- factors based on ALL data will be even larger.

### Fractional atomic coordinates and isotropic or equivalent isotropic displacement parameters (Å^2^)

**Table:**

	*x*	*y*	*z*	*U*_iso_*/*U*_eq_	
Gd1	0.728426 (10)	0.135737 (7)	0.216706 (6)	0.02445 (4)	
Gd2	0.868375 (10)	0.292972 (7)	0.303667 (6)	0.02434 (4)	
O1	0.58635 (16)	0.28209 (10)	0.22392 (11)	0.0379 (4)	
O1W	0.91582 (16)	0.04089 (10)	0.24569 (10)	0.0384 (4)	
H1WA	0.9421	−0.0095	0.2489	0.058*	
H1WB	0.9558	0.0606	0.2606	0.058*	
O2	0.50408 (16)	0.17705 (11)	0.23605 (11)	0.0440 (5)	
O2W	0.67324 (16)	0.38841 (10)	0.28455 (10)	0.0389 (4)	
H2WA	0.6423	0.4419	0.2880	0.058*	
H2WB	0.6263	0.3729	0.2680	0.058*	
O3	0.3780 (2)	0.66676 (12)	0.14719 (11)	0.0576 (6)	
H3	0.3972	0.6761	0.1010	0.086*	
O4	0.69157 (18)	0.16439 (12)	0.08673 (10)	0.0437 (5)	
O5	0.86409 (16)	0.06658 (11)	0.09667 (10)	0.0381 (4)	
O6	0.6696 (2)	−0.23208 (13)	−0.01187 (15)	0.0639 (6)	
H6	0.7274	−0.2699	−0.0385	0.096*	
O7	0.64767 (18)	0.10012 (12)	0.35152 (10)	0.0448 (5)	
O8	0.74284 (15)	0.19881 (10)	0.33397 (9)	0.0302 (4)	
O9	0.83523 (19)	−0.21253 (12)	0.55882 (13)	0.0544 (5)	
H9	0.9098	−0.2293	0.5572	0.082*	
O10	0.85702 (15)	0.23143 (10)	0.18638 (9)	0.0295 (4)	
O11	0.91748 (17)	0.34765 (11)	0.16459 (10)	0.0363 (4)	
O12	0.85826 (18)	0.62580 (11)	−0.08206 (11)	0.0460 (5)	
H12	0.9302	0.6266	−0.1036	0.069*	
O13	1.01650 (15)	0.14806 (10)	0.29342 (11)	0.0376 (4)	
O14	1.09358 (16)	0.25591 (10)	0.25472 (11)	0.0414 (5)	
O15	1.2910 (2)	−0.24392 (11)	0.27918 (11)	0.0532 (5)	
H15	1.3172	−0.2672	0.2375	0.080*	
O16	0.73943 (16)	0.34546 (11)	0.43136 (10)	0.0391 (4)	
O17	0.93402 (18)	0.27265 (12)	0.42362 (10)	0.0461 (5)	
O18	0.6549 (2)	0.71630 (13)	0.55102 (16)	0.0720 (7)	
H18	0.7051	0.7334	0.5629	0.108*	
N1	0.68739 (19)	−0.01014 (13)	0.21714 (12)	0.0332 (5)	
N2	0.6042 (3)	−0.42714 (17)	0.2815 (2)	0.0662 (8)	
N3	0.91443 (19)	0.43674 (12)	0.31052 (11)	0.0319 (5)	
N4	0.9696 (3)	0.86188 (16)	0.2689 (2)	0.0654 (8)	
C1	0.3770 (2)	0.41132 (16)	0.19719 (16)	0.0366 (6)	
C2	0.3614 (3)	0.46773 (17)	0.25268 (16)	0.0422 (7)	
H2A	0.3499	0.4483	0.3031	0.051*	
C3	0.3625 (3)	0.55184 (17)	0.23510 (15)	0.0432 (7)	
H3B	0.3516	0.5886	0.2734	0.052*	
C4	0.3796 (2)	0.58167 (16)	0.16114 (15)	0.0370 (6)	
C5	0.3960 (3)	0.52712 (18)	0.10483 (16)	0.0483 (7)	
H5A	0.4072	0.5470	0.0546	0.058*	
C6	0.3957 (3)	0.44209 (17)	0.12320 (17)	0.0488 (7)	
H6B	0.4085	0.4050	0.0846	0.059*	
C7	0.3744 (3)	0.31993 (17)	0.21737 (19)	0.0482 (7)	
H7A	0.3523	0.2990	0.1779	0.058*	
H7B	0.3081	0.3209	0.2645	0.058*	
C8	0.4950 (2)	0.25578 (16)	0.22693 (14)	0.0335 (6)	
C9	0.7748 (2)	−0.01008 (16)	−0.01699 (14)	0.0348 (6)	
C10	0.8552 (2)	−0.08123 (18)	−0.06146 (16)	0.0428 (7)	
H10A	0.9338	−0.0794	−0.0920	0.051*	
C11	0.8202 (3)	−0.15419 (18)	−0.06094 (17)	0.0463 (7)	
H11A	0.8742	−0.2006	−0.0921	0.056*	
C12	0.7060 (3)	−0.15891 (18)	−0.01472 (16)	0.0429 (7)	
C13	0.6246 (3)	−0.08902 (19)	0.02885 (17)	0.0493 (7)	
H13A	0.5463	−0.0914	0.0594	0.059*	
C14	0.6589 (3)	−0.01520 (18)	0.02744 (16)	0.0437 (7)	
H14A	0.6029	0.0319	0.0570	0.052*	
C15	0.8144 (3)	0.07057 (17)	−0.02025 (14)	0.0404 (6)	
H15B	0.9035	0.0573	−0.0465	0.048*	
H15C	0.7691	0.1159	−0.0491	0.048*	
C16	0.7889 (2)	0.10286 (16)	0.05820 (14)	0.0333 (6)	
C17	0.7278 (3)	0.04549 (17)	0.48684 (14)	0.0411 (7)	
C18	0.8537 (3)	−0.0015 (2)	0.47187 (15)	0.0470 (7)	
H18B	0.9140	0.0252	0.4457	0.056*	
C19	0.8920 (3)	−0.08767 (19)	0.49510 (15)	0.0434 (7)	
H19A	0.9772	−0.1183	0.4844	0.052*	
C20	0.8035 (3)	−0.12784 (17)	0.53414 (15)	0.0388 (6)	
C21	0.6774 (3)	−0.08183 (17)	0.54898 (15)	0.0403 (6)	
H21A	0.6171	−0.1087	0.5749	0.048*	
C22	0.6406 (3)	0.00349 (17)	0.52551 (15)	0.0415 (7)	
H22A	0.5553	0.0336	0.5358	0.050*	
C23	0.6874 (4)	0.13873 (18)	0.46056 (16)	0.0563 (9)	
H23A	0.7420	0.1685	0.4684	0.068*	
H23B	0.6024	0.1673	0.4910	0.068*	
C24	0.6918 (2)	0.14594 (15)	0.37741 (14)	0.0312 (6)	
C25	0.8584 (3)	0.38624 (17)	0.02357 (15)	0.0382 (6)	
C26	0.9606 (3)	0.39378 (18)	−0.03605 (15)	0.0421 (7)	
H26A	1.0296	0.3448	−0.0519	0.051*	
C27	0.9619 (3)	0.47269 (18)	−0.07246 (15)	0.0410 (7)	
H27A	1.0306	0.4765	−0.1130	0.049*	
C28	0.8612 (2)	0.54537 (16)	−0.04844 (14)	0.0352 (6)	
C29	0.7580 (3)	0.53954 (18)	0.01059 (15)	0.0414 (7)	
H29A	0.6894	0.5887	0.0265	0.050*	
C30	0.7572 (3)	0.46032 (18)	0.04574 (15)	0.0422 (7)	
H30A	0.6872	0.4565	0.0852	0.051*	
C31	0.8567 (3)	0.30136 (17)	0.06579 (15)	0.0439 (7)	
H31A	0.7759	0.2931	0.0724	0.053*	
H31B	0.9206	0.2544	0.0345	0.053*	
C32	0.8789 (2)	0.29443 (15)	0.14252 (14)	0.0295 (5)	
C33	1.2555 (2)	0.01976 (15)	0.25513 (14)	0.0312 (6)	
C34	1.3105 (2)	−0.03315 (16)	0.19210 (15)	0.0367 (6)	
H34A	1.3396	−0.0092	0.1444	0.044*	
C35	1.3233 (3)	−0.12107 (17)	0.19864 (15)	0.0407 (7)	
H35A	1.3602	−0.1555	0.1555	0.049*	
C36	1.2812 (2)	−0.15756 (16)	0.26898 (15)	0.0365 (6)	
C37	1.2250 (3)	−0.10546 (17)	0.33231 (15)	0.0395 (6)	
H37A	1.1954	−0.1294	0.3799	0.047*	
C38	1.2129 (2)	−0.01870 (16)	0.32502 (15)	0.0375 (6)	
H38A	1.1751	0.0156	0.3682	0.045*	
C39	1.2429 (2)	0.11494 (15)	0.24847 (16)	0.0365 (6)	
H39A	1.2882	0.1272	0.1969	0.044*	
H39B	1.2835	0.1275	0.2826	0.044*	
C40	1.1101 (2)	0.17600 (15)	0.26615 (14)	0.0301 (6)	
C41	0.7657 (3)	0.44631 (17)	0.54653 (14)	0.0377 (6)	
C42	0.8463 (3)	0.48886 (19)	0.55337 (15)	0.0444 (7)	
H42A	0.9259	0.4563	0.5568	0.053*	
C43	0.8120 (3)	0.57845 (19)	0.55530 (17)	0.0500 (7)	
H43A	0.8682	0.6055	0.5597	0.060*	
C44	0.6939 (3)	0.62779 (18)	0.55074 (16)	0.0455 (7)	
C45	0.6105 (3)	0.58671 (18)	0.54578 (15)	0.0442 (7)	
H45A	0.5300	0.6193	0.5442	0.053*	
C46	0.6470 (3)	0.49732 (17)	0.54315 (15)	0.0406 (6)	
H46A	0.5905	0.4704	0.5390	0.049*	
C47	0.8076 (3)	0.34842 (17)	0.54043 (15)	0.0450 (7)	
H47A	0.8852	0.3240	0.5544	0.054*	
H47B	0.7444	0.3258	0.5761	0.054*	
C48	0.8279 (3)	0.32041 (15)	0.46111 (14)	0.0346 (6)	
C49	0.5820 (2)	−0.02120 (16)	0.26501 (16)	0.0395 (6)	
H49A	0.5218	0.0267	0.2918	0.047*	
C50	0.5573 (3)	−0.09981 (16)	0.27694 (17)	0.0443 (7)	
H50A	0.4827	−0.1043	0.3114	0.053*	
C51	0.6443 (2)	−0.17179 (16)	0.23729 (15)	0.0365 (6)	
C52	0.7519 (3)	−0.16002 (16)	0.18590 (15)	0.0380 (6)	
H52A	0.8119	−0.2063	0.1569	0.046*	
C53	0.7699 (2)	−0.07956 (16)	0.17772 (15)	0.0371 (6)	
H53A	0.8432	−0.0732	0.1430	0.045*	
C54	0.5497 (3)	−0.3656 (2)	0.3339 (2)	0.0725 (11)	
H54A	0.5026	−0.3794	0.3815	0.087*	
C55	0.5587 (3)	−0.28205 (19)	0.3216 (2)	0.0603 (9)	
H55A	0.5198	−0.2418	0.3606	0.072*	
C56	0.6260 (3)	−0.25908 (17)	0.25117 (17)	0.0423 (7)	
C57	0.6791 (4)	−0.3219 (2)	0.1960 (2)	0.0622 (9)	
H57A	0.7233	−0.3093	0.1470	0.075*	
C58	0.6661 (4)	−0.4037 (2)	0.2141 (2)	0.0721 (11)	
H58A	0.7037	−0.4452	0.1761	0.086*	
C59	0.8281 (3)	0.50330 (16)	0.35255 (15)	0.0382 (6)	
H59A	0.7550	0.4942	0.3851	0.046*	
C60	0.8423 (3)	0.58449 (16)	0.34988 (15)	0.0412 (7)	
H60A	0.7799	0.6283	0.3807	0.049*	
C61	0.9487 (3)	0.60137 (15)	0.30181 (15)	0.0359 (6)	
C62	1.0403 (3)	0.53139 (16)	0.26052 (16)	0.0410 (7)	
H62A	1.1153	0.5383	0.2286	0.049*	
C63	1.0199 (2)	0.45177 (16)	0.26701 (15)	0.0375 (6)	
H63A	1.0834	0.4058	0.2394	0.045*	
C64	0.8901 (4)	0.8392 (2)	0.3297 (2)	0.0704 (11)	
H64A	0.8360	0.8820	0.3646	0.084*	
C65	0.8832 (4)	0.75557 (19)	0.34439 (18)	0.0624 (10)	
H65A	0.8272	0.7428	0.3888	0.075*	
C66	0.9601 (3)	0.69054 (17)	0.29244 (17)	0.0426 (7)	
C67	1.0436 (3)	0.71389 (19)	0.2294 (2)	0.0600 (9)	
H67A	1.0981	0.6728	0.1931	0.072*	
C68	1.0455 (3)	0.7992 (2)	0.2207 (3)	0.0719 (11)	
H68A	1.1039	0.8133	0.1783	0.086*	
O3W	0.44251 (19)	0.73311 (13)	0.00868 (11)	0.0560 (5)	
H3WA	0.4028	0.7630	−0.0189	0.084*	
H3WB	0.5018	0.7530	0.0031	0.084*	

### Atomic displacement parameters (Å^2^)

**Table:**

	*U*^11^	*U*^22^	*U*^33^	*U*^12^	*U*^13^	*U*^23^
Gd1	0.02817 (7)	0.01682 (6)	0.03181 (7)	−0.00953 (5)	−0.01159 (5)	0.00229 (5)
Gd2	0.02690 (7)	0.01577 (6)	0.03249 (7)	−0.00774 (5)	−0.01046 (5)	0.00023 (5)
O1	0.0336 (10)	0.0231 (9)	0.0645 (12)	−0.0083 (8)	−0.0242 (9)	−0.0037 (8)
O1W	0.0424 (10)	0.0189 (9)	0.0616 (12)	−0.0085 (8)	−0.0274 (9)	0.0009 (8)
O2	0.0350 (10)	0.0226 (10)	0.0765 (14)	−0.0115 (8)	−0.0193 (9)	0.0091 (9)
O2W	0.0386 (10)	0.0203 (9)	0.0631 (12)	−0.0061 (7)	−0.0244 (9)	−0.0025 (8)
O3	0.0959 (17)	0.0309 (11)	0.0457 (12)	−0.0269 (11)	−0.0122 (12)	−0.0002 (9)
O4	0.0525 (12)	0.0358 (11)	0.0412 (11)	−0.0047 (9)	−0.0190 (9)	−0.0054 (8)
O5	0.0381 (10)	0.0423 (11)	0.0365 (10)	−0.0152 (8)	−0.0113 (8)	−0.0005 (8)
O6	0.0513 (13)	0.0441 (13)	0.0957 (18)	−0.0242 (11)	−0.0054 (12)	−0.0141 (12)
O7	0.0649 (13)	0.0493 (12)	0.0372 (11)	−0.0414 (10)	−0.0154 (10)	0.0057 (9)
O8	0.0354 (9)	0.0214 (9)	0.0364 (10)	−0.0145 (7)	−0.0092 (8)	0.0040 (7)
O9	0.0582 (13)	0.0342 (11)	0.0807 (15)	−0.0142 (10)	−0.0373 (12)	0.0093 (10)
O10	0.0334 (9)	0.0219 (9)	0.0359 (10)	−0.0138 (7)	−0.0103 (8)	0.0059 (7)
O11	0.0501 (11)	0.0329 (10)	0.0377 (10)	−0.0269 (9)	−0.0169 (9)	0.0076 (8)
O12	0.0506 (12)	0.0326 (10)	0.0544 (13)	−0.0190 (9)	−0.0123 (10)	0.0119 (9)
O13	0.0296 (9)	0.0227 (9)	0.0629 (12)	−0.0093 (7)	−0.0144 (9)	−0.0033 (8)
O14	0.0349 (10)	0.0184 (9)	0.0654 (13)	−0.0068 (7)	−0.0093 (9)	0.0020 (8)
O15	0.0790 (15)	0.0256 (10)	0.0578 (13)	−0.0170 (10)	−0.0224 (12)	−0.0010 (9)
O16	0.0402 (10)	0.0376 (11)	0.0429 (11)	−0.0151 (8)	−0.0119 (9)	−0.0041 (8)
O17	0.0501 (12)	0.0410 (11)	0.0415 (11)	0.0015 (9)	−0.0188 (9)	−0.0087 (9)
O18	0.0797 (17)	0.0332 (12)	0.110 (2)	−0.0202 (11)	−0.0326 (15)	−0.0031 (12)
N1	0.0345 (12)	0.0240 (11)	0.0449 (13)	−0.0125 (9)	−0.0135 (10)	0.0008 (9)
N2	0.0628 (18)	0.0322 (15)	0.119 (3)	−0.0242 (14)	−0.0441 (18)	0.0163 (16)
N3	0.0404 (12)	0.0225 (11)	0.0359 (12)	−0.0108 (9)	−0.0141 (10)	−0.0007 (9)
N4	0.082 (2)	0.0296 (15)	0.115 (3)	−0.0256 (15)	−0.068 (2)	0.0154 (16)
C1	0.0265 (13)	0.0261 (14)	0.0595 (18)	−0.0045 (11)	−0.0196 (13)	−0.0001 (12)
C2	0.0502 (17)	0.0346 (15)	0.0404 (16)	−0.0118 (13)	−0.0138 (13)	0.0040 (12)
C3	0.0596 (19)	0.0320 (15)	0.0388 (16)	−0.0130 (13)	−0.0139 (14)	−0.0069 (12)
C4	0.0439 (15)	0.0256 (14)	0.0398 (16)	−0.0086 (11)	−0.0113 (12)	−0.0017 (11)
C5	0.068 (2)	0.0371 (16)	0.0367 (16)	−0.0113 (14)	−0.0153 (14)	−0.0021 (12)
C6	0.065 (2)	0.0297 (15)	0.0522 (18)	−0.0034 (14)	−0.0261 (15)	−0.0110 (13)
C7	0.0398 (16)	0.0291 (15)	0.081 (2)	−0.0091 (12)	−0.0272 (15)	0.0040 (14)
C8	0.0334 (14)	0.0294 (15)	0.0383 (15)	−0.0084 (11)	−0.0128 (12)	0.0011 (11)
C9	0.0411 (15)	0.0362 (15)	0.0317 (14)	−0.0150 (12)	−0.0131 (12)	−0.0012 (11)
C10	0.0325 (15)	0.0439 (17)	0.0487 (17)	−0.0144 (13)	−0.0010 (13)	−0.0097 (13)
C11	0.0381 (16)	0.0387 (16)	0.0575 (19)	−0.0099 (13)	−0.0042 (14)	−0.0150 (13)
C12	0.0393 (16)	0.0401 (16)	0.0534 (18)	−0.0148 (13)	−0.0153 (14)	−0.0033 (13)
C13	0.0348 (15)	0.0531 (19)	0.0560 (19)	−0.0168 (14)	−0.0013 (14)	−0.0076 (15)
C14	0.0398 (16)	0.0415 (16)	0.0450 (17)	−0.0098 (13)	−0.0033 (13)	−0.0139 (13)
C15	0.0507 (17)	0.0399 (16)	0.0325 (15)	−0.0184 (13)	−0.0099 (13)	−0.0001 (12)
C16	0.0412 (15)	0.0291 (14)	0.0343 (14)	−0.0212 (12)	−0.0074 (12)	0.0026 (11)
C17	0.069 (2)	0.0378 (16)	0.0287 (14)	−0.0284 (15)	−0.0196 (14)	0.0038 (11)
C18	0.062 (2)	0.060 (2)	0.0336 (16)	−0.0424 (17)	−0.0117 (14)	0.0041 (13)
C19	0.0433 (16)	0.0516 (18)	0.0402 (16)	−0.0202 (14)	−0.0107 (13)	−0.0071 (13)
C20	0.0488 (17)	0.0343 (15)	0.0417 (16)	−0.0165 (13)	−0.0213 (13)	0.0002 (12)
C21	0.0439 (16)	0.0361 (15)	0.0489 (17)	−0.0237 (13)	−0.0157 (13)	0.0082 (12)
C22	0.0502 (17)	0.0362 (16)	0.0436 (16)	−0.0159 (13)	−0.0200 (14)	0.0041 (12)
C23	0.109 (3)	0.0388 (17)	0.0372 (17)	−0.0405 (18)	−0.0273 (17)	0.0083 (13)
C24	0.0378 (14)	0.0246 (13)	0.0325 (14)	−0.0123 (11)	−0.0104 (11)	0.0031 (10)
C25	0.0566 (18)	0.0360 (15)	0.0346 (15)	−0.0250 (13)	−0.0228 (13)	0.0093 (11)
C26	0.0521 (17)	0.0346 (15)	0.0397 (16)	−0.0127 (13)	−0.0142 (14)	0.0005 (12)
C27	0.0444 (16)	0.0420 (16)	0.0362 (15)	−0.0176 (13)	−0.0084 (13)	0.0045 (12)
C28	0.0433 (15)	0.0333 (15)	0.0380 (15)	−0.0204 (12)	−0.0182 (13)	0.0085 (11)
C29	0.0414 (16)	0.0382 (16)	0.0441 (16)	−0.0134 (13)	−0.0121 (13)	0.0043 (12)
C30	0.0468 (17)	0.0464 (17)	0.0372 (16)	−0.0250 (14)	−0.0096 (13)	0.0090 (13)
C31	0.068 (2)	0.0359 (16)	0.0426 (16)	−0.0304 (14)	−0.0257 (15)	0.0105 (12)
C32	0.0316 (13)	0.0232 (13)	0.0350 (14)	−0.0114 (10)	−0.0098 (11)	0.0037 (10)
C33	0.0258 (13)	0.0257 (13)	0.0409 (15)	−0.0039 (10)	−0.0114 (11)	−0.0026 (11)
C34	0.0395 (15)	0.0301 (14)	0.0337 (14)	−0.0052 (11)	−0.0073 (12)	0.0009 (11)
C35	0.0481 (16)	0.0317 (15)	0.0382 (16)	−0.0034 (12)	−0.0122 (13)	−0.0109 (12)
C36	0.0435 (15)	0.0237 (13)	0.0461 (16)	−0.0096 (11)	−0.0189 (13)	−0.0010 (11)
C37	0.0460 (16)	0.0355 (15)	0.0353 (15)	−0.0138 (12)	−0.0082 (12)	0.0001 (12)
C38	0.0401 (15)	0.0293 (14)	0.0378 (15)	−0.0040 (12)	−0.0077 (12)	−0.0096 (11)
C39	0.0317 (14)	0.0261 (13)	0.0514 (17)	−0.0079 (11)	−0.0115 (12)	−0.0031 (11)
C40	0.0305 (13)	0.0251 (14)	0.0361 (14)	−0.0069 (11)	−0.0114 (11)	−0.0059 (10)
C41	0.0509 (17)	0.0361 (15)	0.0273 (14)	−0.0162 (13)	−0.0087 (12)	−0.0030 (11)
C42	0.0412 (16)	0.0470 (18)	0.0437 (17)	−0.0103 (13)	−0.0106 (13)	−0.0092 (13)
C43	0.0513 (18)	0.0504 (19)	0.0537 (19)	−0.0260 (15)	−0.0095 (15)	−0.0067 (14)
C44	0.0556 (18)	0.0344 (16)	0.0458 (17)	−0.0177 (14)	−0.0086 (14)	−0.0032 (12)
C45	0.0457 (16)	0.0397 (17)	0.0453 (17)	−0.0123 (13)	−0.0121 (13)	0.0014 (13)
C46	0.0446 (16)	0.0419 (17)	0.0377 (15)	−0.0182 (13)	−0.0098 (13)	−0.0009 (12)
C47	0.0660 (19)	0.0344 (16)	0.0335 (15)	−0.0129 (14)	−0.0160 (14)	0.0017 (12)
C48	0.0495 (17)	0.0208 (13)	0.0353 (15)	−0.0143 (12)	−0.0124 (13)	0.0035 (11)
C49	0.0351 (15)	0.0234 (14)	0.0586 (18)	−0.0089 (11)	−0.0104 (13)	−0.0044 (12)
C50	0.0348 (15)	0.0276 (15)	0.070 (2)	−0.0137 (12)	−0.0124 (14)	0.0053 (13)
C51	0.0424 (15)	0.0235 (13)	0.0523 (17)	−0.0134 (12)	−0.0242 (13)	0.0049 (11)
C52	0.0443 (16)	0.0233 (13)	0.0466 (16)	−0.0095 (12)	−0.0131 (13)	−0.0035 (11)
C53	0.0388 (15)	0.0291 (14)	0.0453 (16)	−0.0131 (12)	−0.0124 (13)	0.0017 (12)
C54	0.050 (2)	0.041 (2)	0.116 (3)	−0.0193 (16)	−0.013 (2)	0.024 (2)
C55	0.0447 (18)	0.0318 (16)	0.091 (3)	−0.0124 (14)	−0.0016 (17)	0.0048 (16)
C56	0.0428 (16)	0.0289 (15)	0.066 (2)	−0.0168 (12)	−0.0275 (15)	0.0083 (13)
C57	0.099 (3)	0.0400 (18)	0.064 (2)	−0.0371 (18)	−0.032 (2)	0.0025 (15)
C58	0.113 (3)	0.0343 (18)	0.093 (3)	−0.031 (2)	−0.053 (3)	−0.0005 (18)
C59	0.0455 (16)	0.0277 (14)	0.0398 (15)	−0.0141 (12)	−0.0083 (13)	0.0033 (11)
C60	0.0549 (18)	0.0227 (14)	0.0429 (16)	−0.0082 (12)	−0.0130 (14)	−0.0026 (11)
C61	0.0500 (16)	0.0233 (13)	0.0458 (16)	−0.0148 (12)	−0.0285 (14)	0.0066 (11)
C62	0.0390 (15)	0.0297 (15)	0.0590 (18)	−0.0159 (12)	−0.0168 (14)	0.0047 (13)
C63	0.0378 (15)	0.0260 (14)	0.0496 (17)	−0.0122 (11)	−0.0108 (13)	−0.0017 (11)
C64	0.129 (3)	0.0304 (17)	0.074 (2)	−0.025 (2)	−0.061 (2)	0.0011 (16)
C65	0.118 (3)	0.0331 (17)	0.054 (2)	−0.0319 (18)	−0.043 (2)	0.0039 (14)
C66	0.0564 (18)	0.0263 (14)	0.0613 (19)	−0.0184 (13)	−0.0364 (15)	0.0082 (13)
C67	0.0455 (18)	0.0339 (17)	0.103 (3)	−0.0163 (14)	−0.0243 (18)	0.0114 (17)
C68	0.051 (2)	0.040 (2)	0.133 (3)	−0.0240 (16)	−0.037 (2)	0.027 (2)
O3W	0.0613 (13)	0.0543 (13)	0.0596 (13)	−0.0247 (11)	−0.0270 (11)	0.0189 (10)

### Geometric parameters (Å, °)

**Table:**

Gd1—O10	2.4132 (15)	C17—C23	1.506 (4)
Gd1—O1	2.4146 (16)	C18—C19	1.387 (4)
Gd1—O1W	2.4177 (16)	C18—H18B	0.9300
Gd1—O2	2.4187 (17)	C19—C20	1.379 (4)
Gd1—O5	2.4359 (17)	C19—H19A	0.9300
Gd1—O7	2.4480 (17)	C20—C21	1.381 (4)
Gd1—O4	2.5295 (18)	C21—C22	1.375 (4)
Gd1—O8	2.5671 (16)	C21—H21A	0.9300
Gd1—N1	2.568 (2)	C22—H22A	0.9300
Gd1—C8	2.768 (2)	C23—C24	1.510 (4)
Gd1—C16	2.856 (3)	C23—H23A	0.9700
Gd1—C24	2.875 (2)	C23—H23B	0.9700
Gd2—O8	2.3653 (15)	C25—C30	1.384 (4)
Gd2—O14	2.4075 (17)	C25—C26	1.385 (4)
Gd2—O2W	2.4193 (16)	C25—C31	1.509 (3)
Gd2—O13	2.4207 (16)	C26—C27	1.382 (4)
Gd2—O16	2.4469 (18)	C26—H26A	0.9300
Gd2—O17	2.5118 (18)	C27—C28	1.372 (4)
Gd2—O10	2.5387 (16)	C27—H27A	0.9300
Gd2—O11	2.5697 (16)	C28—C29	1.380 (4)
Gd2—N3	2.5893 (19)	C29—C30	1.377 (4)
Gd2—C40	2.784 (2)	C29—H29A	0.9300
Gd2—C48	2.855 (3)	C30—H30A	0.9300
Gd2—C32	2.932 (2)	C31—C32	1.501 (3)
O1—C8	1.262 (3)	C31—H31A	0.9700
O1W—H1WA	0.7792	C31—H31B	0.9700
O1W—H1WB	0.7713	C33—C34	1.383 (3)
O2—C8	1.246 (3)	C33—C38	1.388 (3)
O2W—H2WA	0.8281	C33—C39	1.501 (3)
O2W—H2WB	0.8199	C34—C35	1.384 (4)
O3—C4	1.374 (3)	C34—H34A	0.9300
O3—H3	0.8200	C35—C36	1.378 (4)
O4—C16	1.262 (3)	C35—H35A	0.9300
O5—C16	1.258 (3)	C36—C37	1.380 (4)
O6—C12	1.379 (3)	C37—C38	1.367 (4)
O6—H6	0.8200	C37—H37A	0.9300
O7—C24	1.238 (3)	C38—H38A	0.9300
O8—C24	1.275 (3)	C39—C40	1.510 (3)
O9—C20	1.369 (3)	C39—H39A	0.9700
O9—H9	0.8200	C39—H39B	0.9700
O10—C32	1.275 (3)	C41—C42	1.381 (4)
O11—C32	1.252 (3)	C41—C46	1.389 (4)
O12—C28	1.377 (3)	C41—C47	1.513 (4)
O12—H12	0.8200	C42—C43	1.382 (4)
O13—C40	1.265 (3)	C42—H42A	0.9300
O14—C40	1.254 (3)	C43—C44	1.382 (4)
O15—C36	1.368 (3)	C43—H43A	0.9300
O15—H15	0.8200	C44—C45	1.381 (4)
O16—C48	1.253 (3)	C45—C46	1.378 (4)
O17—C48	1.265 (3)	C45—H45A	0.9300
O18—C44	1.362 (3)	C46—H46A	0.9300
O18—H18	0.8200	C47—C48	1.506 (4)
N1—C49	1.335 (3)	C47—H47A	0.9700
N1—C53	1.336 (3)	C47—H47B	0.9700
N2—C58	1.316 (5)	C49—C50	1.380 (3)
N2—C54	1.329 (5)	C49—H49A	0.9300
N3—C63	1.333 (3)	C50—C51	1.382 (4)
N3—C59	1.340 (3)	C50—H50A	0.9300
N4—C64	1.323 (5)	C51—C52	1.382 (4)
N4—C68	1.324 (5)	C51—C56	1.487 (3)
C1—C6	1.375 (4)	C52—C53	1.377 (3)
C1—C2	1.382 (4)	C52—H52A	0.9300
C1—C7	1.499 (4)	C53—H53A	0.9300
C2—C3	1.373 (4)	C54—C55	1.385 (4)
C2—H2A	0.9300	C54—H54A	0.9300
C3—C4	1.371 (4)	C55—C56	1.379 (4)
C3—H3B	0.9300	C55—H55A	0.9300
C4—C5	1.370 (4)	C56—C57	1.378 (4)
C5—C6	1.387 (4)	C57—C58	1.381 (4)
C5—H5A	0.9300	C57—H57A	0.9300
C6—H6B	0.9300	C58—H58A	0.9300
C7—C8	1.510 (3)	C59—C60	1.378 (4)
C7—H7A	0.9700	C59—H59A	0.9300
C7—H7B	0.9700	C60—C61	1.381 (4)
C9—C14	1.379 (4)	C60—H60A	0.9300
C9—C10	1.391 (4)	C61—C62	1.390 (4)
C9—C15	1.517 (4)	C61—C66	1.485 (3)
C10—C11	1.374 (4)	C62—C63	1.378 (3)
C10—H10A	0.9300	C62—H62A	0.9300
C11—C12	1.372 (4)	C63—H63A	0.9300
C11—H11A	0.9300	C64—C65	1.381 (4)
C12—C13	1.371 (4)	C64—H64A	0.9300
C13—C14	1.380 (4)	C65—C66	1.389 (4)
C13—H13A	0.9300	C65—H65A	0.9300
C14—H14A	0.9300	C66—C67	1.377 (4)
C15—C16	1.503 (3)	C67—C68	1.385 (4)
C15—H15B	0.9700	C67—H67A	0.9300
C15—H15C	0.9700	C68—H68A	0.9300
C17—C18	1.383 (4)	O3W—H3WA	0.8095
C17—C22	1.385 (4)	O3W—H3WB	0.8339
			
O10—Gd1—O1	73.49 (5)	C12—C11—H11A	119.9
O10—Gd1—O1W	79.74 (5)	C10—C11—H11A	119.9
O1—Gd1—O1W	145.42 (6)	C13—C12—C11	119.6 (3)
O10—Gd1—O2	126.77 (5)	C13—C12—O6	118.9 (3)
O1—Gd1—O2	53.62 (6)	C11—C12—O6	121.5 (3)
O1W—Gd1—O2	149.95 (6)	C12—C13—C14	120.1 (3)
O10—Gd1—O5	84.86 (6)	C12—C13—H13A	120.0
O1—Gd1—O5	123.03 (6)	C14—C13—H13A	120.0
O1W—Gd1—O5	74.79 (6)	C9—C14—C13	121.2 (3)
O2—Gd1—O5	117.09 (6)	C9—C14—H14A	119.4
O10—Gd1—O7	117.15 (6)	C13—C14—H14A	119.4
O1—Gd1—O7	95.22 (7)	C16—C15—C9	111.5 (2)
O1W—Gd1—O7	77.79 (6)	C16—C15—H15B	109.3
O2—Gd1—O7	76.98 (7)	C9—C15—H15B	109.3
O5—Gd1—O7	140.79 (6)	C16—C15—H15C	109.3
O10—Gd1—O4	90.62 (6)	C9—C15—H15C	109.3
O1—Gd1—O4	75.77 (6)	H15B—C15—H15C	108.0
O1W—Gd1—O4	126.62 (6)	O5—C16—O4	119.6 (2)
O2—Gd1—O4	72.53 (6)	O5—C16—C15	119.5 (2)
O5—Gd1—O4	51.99 (6)	O4—C16—C15	120.9 (2)
O7—Gd1—O4	147.45 (6)	O5—C16—Gd1	58.02 (13)
O10—Gd1—O8	66.00 (5)	O4—C16—Gd1	62.29 (13)
O1—Gd1—O8	75.82 (5)	C15—C16—Gd1	169.50 (17)
O1W—Gd1—O8	73.47 (5)	C18—C17—C22	117.8 (3)
O2—Gd1—O8	102.56 (6)	C18—C17—C23	120.9 (3)
O5—Gd1—O8	139.94 (5)	C22—C17—C23	121.3 (3)
O7—Gd1—O8	51.47 (5)	C17—C18—C19	121.4 (3)
O4—Gd1—O8	147.34 (5)	C17—C18—H18B	119.3
O10—Gd1—N1	154.44 (6)	C19—C18—H18B	119.3
O1—Gd1—N1	130.04 (6)	C20—C19—C18	119.8 (3)
O1W—Gd1—N1	81.33 (6)	C20—C19—H19A	120.1
O2—Gd1—N1	76.56 (6)	C18—C19—H19A	120.1
O5—Gd1—N1	73.68 (6)	O9—C20—C19	122.6 (3)
O7—Gd1—N1	74.93 (6)	O9—C20—C21	118.0 (2)
O4—Gd1—N1	87.01 (6)	C19—C20—C21	119.4 (3)
O8—Gd1—N1	123.99 (6)	C22—C21—C20	120.2 (3)
O10—Gd1—C8	100.05 (7)	C22—C21—H21A	119.9
O1—Gd1—C8	27.11 (6)	C20—C21—H21A	119.9
O1W—Gd1—C8	163.63 (7)	C21—C22—C17	121.4 (3)
O2—Gd1—C8	26.73 (6)	C21—C22—H22A	119.3
O5—Gd1—C8	121.57 (7)	C17—C22—H22A	119.3
O7—Gd1—C8	87.98 (7)	C17—C23—C24	111.9 (2)
O4—Gd1—C8	69.67 (7)	C17—C23—H23A	109.2
O8—Gd1—C8	91.37 (6)	C24—C23—H23A	109.2
N1—Gd1—C8	102.93 (7)	C17—C23—H23B	109.2
O10—Gd1—C16	89.89 (6)	C24—C23—H23B	109.2
O1—Gd1—C16	100.66 (7)	H23A—C23—H23B	107.9
O1W—Gd1—C16	100.77 (7)	O7—C24—O8	120.4 (2)
O2—Gd1—C16	93.83 (7)	O7—C24—C23	120.3 (2)
O5—Gd1—C16	25.99 (6)	O8—C24—C23	119.3 (2)
O7—Gd1—C16	151.73 (6)	O7—C24—Gd1	57.67 (13)
O4—Gd1—C16	26.22 (6)	O8—C24—Gd1	63.23 (12)
O8—Gd1—C16	155.75 (6)	C23—C24—Gd1	171.66 (19)
N1—Gd1—C16	76.94 (7)	C30—C25—C26	117.9 (2)
C8—Gd1—C16	95.60 (8)	C30—C25—C31	120.0 (3)
O10—Gd1—C24	91.87 (6)	C26—C25—C31	122.1 (3)
O1—Gd1—C24	87.13 (7)	C27—C26—C25	121.2 (3)
O1W—Gd1—C24	72.09 (7)	C27—C26—H26A	119.4
O2—Gd1—C24	91.21 (7)	C25—C26—H26A	119.4
O5—Gd1—C24	146.77 (6)	C28—C27—C26	119.6 (3)
O7—Gd1—C24	25.30 (6)	C28—C27—H27A	120.2
O4—Gd1—C24	161.24 (6)	C26—C27—H27A	120.2
O8—Gd1—C24	26.31 (6)	C27—C28—O12	122.0 (2)
N1—Gd1—C24	98.49 (7)	C27—C28—C29	120.3 (2)
C8—Gd1—C24	91.59 (7)	O12—C28—C29	117.7 (2)
C16—Gd1—C24	172.19 (7)	C30—C29—C28	119.5 (3)
O8—Gd2—O14	128.23 (5)	C30—C29—H29A	120.3
O8—Gd2—O2W	78.66 (6)	C28—C29—H29A	120.3
O14—Gd2—O2W	143.57 (6)	C29—C30—C25	121.4 (3)
O8—Gd2—O13	75.12 (5)	C29—C30—H30A	119.3
O14—Gd2—O13	53.70 (6)	C25—C30—H30A	119.3
O2W—Gd2—O13	147.68 (6)	C32—C31—C25	115.0 (2)
O8—Gd2—O16	81.01 (6)	C32—C31—H31A	108.5
O14—Gd2—O16	126.83 (6)	C25—C31—H31A	108.5
O2W—Gd2—O16	75.76 (6)	C32—C31—H31B	108.5
O13—Gd2—O16	117.69 (6)	C25—C31—H31B	108.5
O8—Gd2—O17	98.64 (6)	H31A—C31—H31B	107.5
O14—Gd2—O17	77.82 (6)	O11—C32—O10	119.4 (2)
O2W—Gd2—O17	127.09 (6)	O11—C32—C31	122.7 (2)
O13—Gd2—O17	75.88 (6)	O10—C32—C31	117.9 (2)
O16—Gd2—O17	52.01 (6)	O11—C32—Gd2	60.97 (12)
O8—Gd2—O10	67.13 (5)	O10—C32—Gd2	59.64 (12)
O14—Gd2—O10	90.95 (6)	C31—C32—Gd2	168.77 (18)
O2W—Gd2—O10	76.95 (6)	C34—C33—C38	117.4 (2)
O13—Gd2—O10	75.66 (6)	C34—C33—C39	121.4 (2)
O16—Gd2—O10	141.35 (5)	C38—C33—C39	121.2 (2)
O17—Gd2—O10	150.66 (5)	C33—C34—C35	121.2 (2)
O8—Gd2—O11	115.28 (5)	C33—C34—H34A	119.4
O14—Gd2—O11	73.16 (6)	C35—C34—H34A	119.4
O2W—Gd2—O11	72.69 (6)	C36—C35—C34	120.0 (2)
O13—Gd2—O11	101.96 (6)	C36—C35—H35A	120.0
O16—Gd2—O11	140.12 (6)	C34—C35—H35A	120.0
O17—Gd2—O11	144.49 (6)	O15—C36—C35	122.8 (2)
O10—Gd2—O11	50.57 (5)	O15—C36—C37	117.8 (2)
O8—Gd2—N3	154.59 (6)	C35—C36—C37	119.4 (2)
O14—Gd2—N3	75.97 (6)	C38—C37—C36	119.9 (2)
O2W—Gd2—N3	83.92 (6)	C38—C37—H37A	120.0
O13—Gd2—N3	126.60 (6)	C36—C37—H37A	120.0
O16—Gd2—N3	76.84 (6)	C37—C38—C33	122.0 (2)
O17—Gd2—N3	77.34 (6)	C37—C38—H38A	119.0
O10—Gd2—N3	126.51 (6)	C33—C38—H38A	119.0
O11—Gd2—N3	76.15 (6)	C33—C39—C40	115.8 (2)
O8—Gd2—C40	101.90 (6)	C33—C39—H39A	108.3
O14—Gd2—C40	26.70 (6)	C40—C39—H39A	108.3
O2W—Gd2—C40	157.94 (7)	C33—C39—H39B	108.3
O13—Gd2—C40	27.00 (6)	C40—C39—H39B	108.3
O16—Gd2—C40	126.27 (7)	H39A—C39—H39B	107.4
O17—Gd2—C40	74.87 (6)	O14—C40—O13	119.9 (2)
O10—Gd2—C40	82.95 (6)	O14—C40—C39	118.8 (2)
O11—Gd2—C40	87.57 (7)	O13—C40—C39	121.3 (2)
N3—Gd2—C40	101.18 (7)	O14—C40—Gd2	59.66 (12)
O8—Gd2—C48	91.76 (6)	O13—C40—Gd2	60.29 (12)
O14—Gd2—C48	101.95 (7)	C39—C40—Gd2	177.10 (18)
O2W—Gd2—C48	100.94 (7)	C42—C41—C46	117.2 (2)
O13—Gd2—C48	98.45 (7)	C42—C41—C47	120.9 (3)
O16—Gd2—C48	25.90 (7)	C46—C41—C47	121.8 (3)
O17—Gd2—C48	26.27 (6)	C41—C42—C43	121.8 (3)
O10—Gd2—C48	158.86 (6)	C41—C42—H42A	119.1
O11—Gd2—C48	149.45 (6)	C43—C42—H42A	119.1
N3—Gd2—C48	73.45 (7)	C44—C43—C42	119.9 (3)
C40—Gd2—C48	101.09 (7)	C44—C43—H43A	120.1
O8—Gd2—C32	90.56 (6)	C42—C43—H43A	120.1
O14—Gd2—C32	83.98 (7)	O18—C44—C45	117.8 (3)
O2W—Gd2—C32	70.21 (6)	O18—C44—C43	122.8 (3)
O13—Gd2—C32	91.34 (6)	C45—C44—C43	119.4 (3)
O16—Gd2—C32	145.93 (6)	C46—C45—C44	119.8 (3)
O17—Gd2—C32	161.63 (6)	C46—C45—H45A	120.1
O10—Gd2—C32	25.68 (6)	C44—C45—H45A	120.1
O11—Gd2—C32	25.21 (6)	C45—C46—C41	121.8 (3)
N3—Gd2—C32	100.84 (6)	C45—C46—H46A	119.1
C40—Gd2—C32	87.73 (7)	C41—C46—H46A	119.1
C48—Gd2—C32	170.20 (7)	C48—C47—C41	111.9 (2)
C8—O1—Gd1	92.24 (14)	C48—C47—H47A	109.2
Gd1—O1W—H1WA	132.3	C41—C47—H47A	109.2
Gd1—O1W—H1WB	119.3	C48—C47—H47B	109.2
H1WA—O1W—H1WB	107.7	C41—C47—H47B	109.2
C8—O2—Gd1	92.46 (15)	H47A—C47—H47B	107.9
Gd2—O2W—H2WA	130.6	O16—C48—O17	119.5 (2)
Gd2—O2W—H2WB	124.4	O16—C48—C47	119.9 (2)
H2WA—O2W—H2WB	104.7	O17—C48—C47	120.6 (2)
C4—O3—H3	109.5	O16—C48—Gd2	58.53 (13)
C16—O4—Gd1	91.49 (15)	O17—C48—Gd2	61.53 (13)
C16—O5—Gd1	95.99 (15)	C47—C48—Gd2	171.38 (17)
C12—O6—H6	109.5	N1—C49—C50	123.6 (2)
C24—O7—Gd1	97.02 (15)	N1—C49—H49A	118.2
C24—O8—Gd2	153.55 (16)	C50—C49—H49A	118.2
C24—O8—Gd1	90.46 (14)	C49—C50—C51	119.4 (3)
Gd2—O8—Gd1	113.76 (6)	C49—C50—H50A	120.3
C20—O9—H9	109.5	C51—C50—H50A	120.3
C32—O10—Gd1	142.46 (15)	C50—C51—C52	117.2 (2)
C32—O10—Gd2	94.68 (14)	C50—C51—C56	121.7 (3)
Gd1—O10—Gd2	113.11 (6)	C52—C51—C56	121.1 (2)
C32—O11—Gd2	93.81 (14)	C53—C52—C51	119.8 (2)
C28—O12—H12	109.5	C53—C52—H52A	120.1
C40—O13—Gd2	92.71 (13)	C51—C52—H52A	120.1
C40—O14—Gd2	93.64 (15)	N1—C53—C52	123.3 (3)
C36—O15—H15	109.5	N1—C53—H53A	118.4
C48—O16—Gd2	95.57 (15)	C52—C53—H53A	118.4
C48—O17—Gd2	92.20 (15)	N2—C54—C55	123.9 (3)
C44—O18—H18	109.5	N2—C54—H54A	118.1
C49—N1—C53	116.6 (2)	C55—C54—H54A	118.1
C49—N1—Gd1	118.59 (16)	C56—C55—C54	119.4 (3)
C53—N1—Gd1	124.45 (17)	C56—C55—H55A	120.3
C58—N2—C54	115.9 (3)	C54—C55—H55A	120.3
C63—N3—C59	116.2 (2)	C57—C56—C55	116.9 (3)
C63—N3—Gd2	121.96 (16)	C57—C56—C51	122.1 (3)
C59—N3—Gd2	121.48 (17)	C55—C56—C51	121.0 (3)
C64—N4—C68	116.5 (3)	C56—C57—C58	119.3 (3)
C6—C1—C2	117.5 (2)	C56—C57—H57A	120.4
C6—C1—C7	121.6 (3)	C58—C57—H57A	120.4
C2—C1—C7	120.9 (3)	N2—C58—C57	124.6 (3)
C3—C2—C1	121.5 (3)	N2—C58—H58A	117.7
C3—C2—H2A	119.3	C57—C58—H58A	117.7
C1—C2—H2A	119.3	N3—C59—C60	123.2 (3)
C4—C3—C2	120.1 (3)	N3—C59—H59A	118.4
C4—C3—H3B	119.9	C60—C59—H59A	118.4
C2—C3—H3B	119.9	C59—C60—C61	120.5 (2)
C5—C4—C3	119.7 (2)	C59—C60—H60A	119.7
C5—C4—O3	122.8 (2)	C61—C60—H60A	119.7
C3—C4—O3	117.5 (2)	C60—C61—C62	116.2 (2)
C4—C5—C6	119.6 (3)	C60—C61—C66	121.2 (2)
C4—C5—H5A	120.2	C62—C61—C66	122.5 (3)
C6—C5—H5A	120.2	C63—C62—C61	119.9 (3)
C1—C6—C5	121.5 (3)	C63—C62—H62A	120.1
C1—C6—H6B	119.2	C61—C62—H62A	120.1
C5—C6—H6B	119.2	N3—C63—C62	123.9 (2)
C1—C7—C8	115.9 (2)	N3—C63—H63A	118.1
C1—C7—H7A	108.3	C62—C63—H63A	118.1
C8—C7—H7A	108.3	N4—C64—C65	123.6 (4)
C1—C7—H7B	108.3	N4—C64—H64A	118.2
C8—C7—H7B	108.3	C65—C64—H64A	118.2
H7A—C7—H7B	107.4	C64—C65—C66	119.7 (3)
O2—C8—O1	120.7 (2)	C64—C65—H65A	120.1
O2—C8—C7	119.5 (2)	C66—C65—H65A	120.1
O1—C8—C7	119.8 (2)	C67—C66—C65	116.7 (3)
O2—C8—Gd1	60.81 (13)	C67—C66—C61	121.3 (3)
O1—C8—Gd1	60.65 (12)	C65—C66—C61	122.0 (3)
C7—C8—Gd1	169.9 (2)	C66—C67—C68	119.3 (3)
C14—C9—C10	117.8 (2)	C66—C67—H67A	120.3
C14—C9—C15	122.3 (2)	C68—C67—H67A	120.3
C10—C9—C15	119.8 (2)	N4—C68—C67	124.1 (4)
C11—C10—C9	121.0 (3)	N4—C68—H68A	117.9
C11—C10—H10A	119.5	C67—C68—H68A	117.9
C9—C10—H10A	119.5	H3WA—O3W—H3WB	104.8
C12—C11—C10	120.3 (3)		

### Hydrogen-bond geometry (Å, °)

**Table:**

*D*—H···*A*	*D*—H	H···*A*	*D*···*A*	*D*—H···*A*
O1W—H1WB···O13	0.77	2.00	2.744 (2)	161
O1W—H1WA···N4^i^	0.78	2.02	2.784 (3)	167
O2W—H2WB···O1	0.82	2.00	2.750 (2)	151
O2W—H2WA···N2^ii^	0.83	2.03	2.842 (3)	167
O3—H3···O3W	0.82	1.86	2.641 (3)	160
O6—H6···O12^i^	0.82	1.94	2.743 (3)	168
O9—H9···O17^iii^	0.82	1.87	2.675 (3)	167
O12—H12···O11^iv^	0.82	1.94	2.750 (3)	167
O15—H15···O3^v^	0.82	1.90	2.717 (3)	174
O18—H18···O9^ii^	0.82	1.97	2.767 (3)	162
O3W—H3WA···O4^vi^	0.81	1.96	2.773 (3)	179
O3W—H3WB···O6^ii^	0.83	1.99	2.808 (3)	165

Symmetry codes: (i) *x*, *y*−1, *z*; (ii) *x*, *y*+1, *z*; (iii) −*x*+2, −*y*, −*z*+1; (iv) −*x*+2, −*y*+1, −*z*; (v) *x*+1, *y*−1, *z*; (vi) −*x*+1, −*y*+1, −*z*.
